# Association of age with class VI composite restoration

**DOI:** 10.6026/973206300161094

**Published:** 2020-12-31

**Authors:** Sneha Pai, Delphine Priscilla Antony, Adimulapu Hima Sandeep

**Affiliations:** 1 Saveetha Dental College, Saveetha Institute of Medical and Technical Sciences, Saveetha University, Chennai 77, India

**Keywords:** Class VI caries lesion, direct restoration technique, cusp build up, adhesive tooth repair, indirect restorations

## Abstract

Dental caries is the major oral health problem in most of the countries, affecting 60-90% of school children and a vast majority of adults. Therefore, it is of interest to evaluate the association of age with Class VI defects restored with composite restorations. We used 102 cases with data regarding Class VI composite restorations in a datasheet of 86,000 records at Saveetha Dental College, India for this study. Data shows that Class VI restorations were commonly seen in upper anterior teeth in the age group of 51 and above. The cavities prepared to receive Class VI restoration followed a conservative design of caries removal and used direct restoration techniques for reconstruction of the lost tooth structure.

## Background:

Dental caries and trauma (which includes tooth fractures, avulsion as well as non carious lesions) are the two most common conditions leading to pulpal inflammation [1]. Dental caries is defined as an infectious microbiological disease of the teeth that results in localised dissolution and destruction of the calcified tissues [2]. Black's classification of caries lesion had only 5 classes. Simon added a new class to this classification. The Class VI lesions were described as those occurring at the incisal edge or occlusal cusps due to attrition, abrasion or erosion [3]. This Class VI lesion was also referred as "Simon's modification" [4]. Class VI enamel defects of enamel are usually seen in older, worn out teeth in which enamel layer has been lost resulting in exposure of low mineralized dentin which is susceptible to wear, acid content within saliva and food, and caries. Class VI caries lesion of posterior teeth usually occurs on the cusp tips which are usually sites due to the absence of obvious patencies like pits and fissures that serve as areas for biofilm formation and plaque accumulation. Therefore the etiology remains unknown [5]. Few authors believe that microscopic porosities develop at the cusp tips or the incisal edges naturally or due to masticatory stresses, which promote microbial colonization and caries progression. As the caries lesion develops, the area gets further demineralised and results in breakdown of tooth structure [6]. So far, various materials like gold foil, silver amalgam, resin based composites and ceramics have been used to repair Class VI defects [3,^7^]. Therefore, it is of interest to evaluate the association of age with Class VI defects restored with composite restorations.

## Methods:

Ethical approval: Approval for the project was obtained from the Institutional Review Board of Saveetha Institute of Medical and Technical Sciences, Chennai, India on 24/4/2020. SDC/SIHEC/2020/DIASDATA/0619-0320. A university set up was selected for this study, which provided easy accessibility to data of a population of similar ethnicity. There were 2 reviewers to analyze the data retrieved for the study. Data was retrieved of 86,000 patients between June 2019 and March 2020 from the patient records of a private dental college, which was then analyzed. All those Class VI lesion restored with composite were included for this study. Class VI amalgam and GIC restored cases were excluded. Duplicate entries were removed and the data was copied to SPSS software. After entering the data in SPSS software, the variables were verified and frequency distribution tables were prepared. Association of age and gender with cavity design and type of restoration for Class VI composite cases was evaluated using chi square test.

## Results and Discussion:

The parameters assessed in this study were age and gender of the individual, cavity designed and type of restoration for Class VI cases. The retrieved data had 102 cases of Class VI lesions that were restored using composite. Association was done between age and cavity design and type of restoration using chi square test which showed statistical significant difference between age and Class VI composite restorations.[p value < 0.05 (chi square test)] . In this study association of age with Class VI composite restorations was evaluated by associating age with cavity design and type of restoration used for Class VI cases. When the frequency of placement of Class VI restorations was studied, it showed that most of the cases belonged to the age group of 51 and above, involving teeth of sextant 2 (#13-23) where a conservative design of carious removal was followed prior to composite restoration. When association was done between age and cavity design a statistical significant difference was seen with cusp build up being followed in most of the individuals aged 51 and above and caries removal in the young age group (Figure 1 - see PDF). This finding showed cusp build up in older individuals is probably because of the excessive attrition leading to fracture to cusp or development of Class VI lesion. The conservative approach was followed more among younger individuals as localized caries lesion was seen [8]. On correlating tooth number and cavity design, no significant difference was seen. (Figure 2 - see PDF) Sextant 2 (#13-23) was seen to be commonly involved with Class VI lesions and both caries removal and cusp build up was equally practiced prior to composite restoration. On associating age and tooth number with the type of Class VI restoration, frequency distribution tables were studied which showed most of the Class VI cases having been restored with direct restoration technique in comparison to bilayered technique. A significant difference was seen when the age and type of restoration were associated with most of the cases being restored with direct restoration over bilayered restoration (Figure 3 - see PDF). Direct restoration technique was more commonly followed in younger and older age groups with bilayered restoration being practiced more in the middle age group. The association between age and tooth number showed no significant difference with both direct restoration and bilayered restorations being practiced almost equally among all teeth (Figure 4 - see PDF). The practice of direct restoration is more commonly seen in young age groups as they are aware and conscious about dental esthetics which makes them approach the dentist during early stages of caries which can be managed without pulp protection [9]. In older individuals, presence of increased sclerotic dentin, dead tracts, formation of reparative and reactive dentin would all protect the pulp from the progressing dental caries. This makes restorations possible without pulp protection in most of the older individuals [10]. With the developing dental awareness, tooth structure loss these days is caused more due to physiologic changes in the tooth than dental caries [11]. Advancements in restorative dentistry have led to the use of additive techniques over subtractive approaches for restoring lost tooth structures [12]. Occlusal loss of tooth structure resulting in wearing away or fracture of the cusps can also be considered as a Class VI lesion. Such cases, resulting in minimal loss of tooth structure can be restored using composite resins. Direct composite build ups for the restoration of moderate to large Class VI defects and for cusp build up of posterior teeth have shown deterioration in shape, fit and surface texture of restorations [13]. With the position of the tooth being unfavorable in case of molars, direct restoration of moderate to large tooth structure loss has shown poor survival rates and in such cases, for re establishment of tooth structure indirect restorations with onlays have been proposed [14]. These indirect restorations along with minimal reduction of hard tissue, provides a stable and physiologic occlusion along with optimal form and esthetics [15]. Therefore, prior to restoring a Class VI defect, a thorough clinical examination and assessment of the defect is necessary which would further dictate the type of preparation and the restorative technique.

## Conclusion

This study showed statistical significant difference in the age and Class VI composite restorations with the most of the Class VI lesions occurring in the age group of 51 and above. Many of the Class VI lesions were seen in upper anterior teeth and were managed by carious removal and direct restorative technique using composite resins. This study was carried out with a small sample size, which made generalization of the results impossible. Further studies can be carried out with larger sample size and by evaluating the durability of these Class VI composite restorations. The study results revealed a statistically significant difference in the association of age with Class VI composite restorations. It also showed that the cavity design and the type of restoration depend on the extent of decay. Further studies have to be carried out to evaluate the durability of such restorations.
